# Near-Infrared Fluorescence Detection of Acetylcholine in Aqueous Solution Using a Complex of Rhodamine 800 and *p*-Sulfonato-calix[8]arene

**DOI:** 10.3390/s100302438

**Published:** 2010-03-23

**Authors:** Takashi Jin

**Affiliations:** WPI Immunology Frontier Research Center, Osaka University, Yamada-oka, 1-3, Suita, Osaka 565-0871, Japan; E-Mail: jin@fbs.osaka-u.ac.jp; Tel.: +81-6-6879-4427; Fax: +81-6-6879-4426

**Keywords:** near-infrared fluorescence, fluorescence detection, acetylcholine, dopamine, rhodamine 800, *p*-sulfonatocalixarene

## Abstract

The complexing properties of *p*-sulfonatocalix[n]arenes (n = 4: S[4], n = 6: S[6], and n = 8: S[8]) for rhodamine 800 (Rh800) and indocyanine green (ICG) were examined to develop a near-infrared (NIR) fluorescence detection method for acetylcholine (ACh). We found that Rh800 (as a cation) forms an inclusion complex with S[n], while ICG (as a twitter ion) have no binding ability for S[n]. The binding ability of Rh800 to S[n] decreased in the order of S[8] > S[6] >> S[4]. By the formation of the complex between Rh800 and S[8], fluorescence intensity of the Rh800 was significantly decreased. From the fluorescence titration of Rh800 by S[8], stoichiometry of the Rh800-S[8] complex was determined to be 1:1 with a dissociation constant of 2.2 μM in PBS. The addition of ACh to the aqueous solution of the Rh800-S[8] complex caused a fluorescence increase of Rh800, resulting from a competitive replacement of Rh800 by ACh in the complex. From the fluorescence change by the competitive fluorophore replacement, stoichiometry of the Rh800-ACh complex was found to be 1:1 with a dissociation constant of 1.7 mM. The effects of other neurotransmitters on the fluorescence spectra of the Rh800-S[8] complex were examined for dopamine, GABA, glycine, and *l*-asparatic acid. Among the neurotransmitters examined, fluorescence response of the Rh800-S[8] complex was highly specific to ACh. Rh800-S[8] complexes can be used as a NIR fluorescent probe for the detection of ACh (5 × 10^−4^−10^−3^ M) in PBS buffer (pH = 7.2).

## 1. Introduction

Acetylcholine (ACh) is one of the most abundant neurotransmitters in the nervous system [1]. ACh acts as a transmitter at synapses in the ganglia of the visceral motor system, and at a variety of sites within the central nervous system. After the binding to ACh receptors at postsynaptic membranes, ACh is rapidly hydrolyzed to choline and acetic acids by ACh esterase to prevent over-accumulation of ACh. The concentration of ACh in the synaptic cleft of an active neuromuscular junction is about 5 × 10^−4^ M [2]. There are many methods for measuring the concentration of ACh: GC-MS [3] and HPLC with chemiluminescence assay [4,5], colorimetric assay [6], and radioimmunoassay [7,8]. Recently, electrochemical methods using ACh esterase-modified electrodes have been developed [9,10]. However, these methods involve some complicated and time-consuming procedures, and require expensive equipments. The objective of this work is to develop a facile fluorescence detection method for ACh in the near-infrared (NIR) region.

Compared to optical sensing using absorption, fluorescence sensing is simple and highly sensitive, because fluorescence intensity is measured directly without comparison with a reference [11]. By using a conventional spectrofluorometer, the concentration (μM−nM) of fluorescent molecules in aqueous solution can be easily determined. However, fluorescence sensing in biological samples has some complicated problem concerning light absorption and scattering. For example, in living tissues, there are many intrinsic chromophores like hemoproteins and flavin-containing proteins that absorb or emit visible light [11,12]. In contrast, NIR light ranging from 700 nm to 1100 nm is useful for *in vivo* fluorescence sensing. In living tissues, the absorption coefficient of NIR light is much lower than that of visible light, and the scattering of NIR light is much smaller than that of visible light [13,14].

In this paper, we report a NIR fluorescence detection method for ACh using a complex of Rh800 and S[8]. So far, it has been shown that S[n] have binding abilities for quaternary ammonium cations [15–19], and they are able to recognize ACh in aqueous solution [20–27]. Koh *et al.* first reported the utility of the complex of S[6] and a fluorescent ACh analog for the detection of ACh in aqueous solution [21]. Previously, we reported that S[8] strongly binds to fluorescent ACh analogs with cationic charges, dansylcholine [23] and rhodamine 6G [27] in aqueous solution, and their fluorescence can be used for the detection of 10^−4^ M levels of ACh in aqueous solution. Recently, Korbakov *et al.* [26] have reported ACh detection at micromolar concentrations by use of the complex between a cationic fluorescent guest, *trans*-4-[4-(dimethylamino)styryl]-1-methylpyridium *p*-toluensulfonate with S[6]. All of these methods, however, are limited to fluorescence detection of ACh in the visible region. To the best of our knowledge, there are no reports of NIR fluorescent probes for detecting ACh in aqueous solutions.

**Scheme 1.**
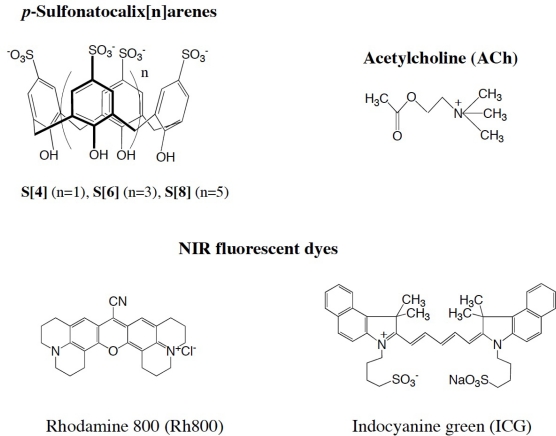
Molecular structure of the *p*-sulfonatocalix[n]arenes (S[n]): acetylcholine (ACh), and the NIR-fluorescent dyes: rhodamine 800 (Rh800) and indocyanine green (ICG).

To develop a NIR fluorescence detection method for ACh, we examined complexing properties of NIR fluorescent dyes (Rh800 and ICG) for S [n] in aqueous solution (Scheme 1). We found that Rh800 has a strong binding ability to S[8], while ICG shows no binding ability to S[n]. The formation of Rh800-S[n] complex and the competitive binding of ACh to Rh800-S[8] complex were examined by fluorescence titration experiments. The binding selectivity of the Rh800-S[8] complex was quite specific to ACh among other neurotransmitters such as dopamine, GABA, and glycine. We demonstrate the utility of Rh800-S[8] complex as a NIR fluorescent probe for determining ACh concentration (5 × 10^−4^−10^−3^ M) in aqueous solutions.

## 2. Results and Discussion

### 2.1. Fluorescence Properties of Rh800 and ICG

We have chosen Rh800 and ICG as guest NIR fluorophores for S[n]. Rh800 is one of the few rhodamines that have NIR fluorescence, and it has been used as a laser dye [28]. Recently, Rh800 was also used as a mitochondrial membrane potential sensitive dye [29]. ICG is a most popular NIR dye and is used for medical diagnostics such as hepatic function, liver blood flow, and for ophthalmic angiography [30,31]. Rh800 has a cationic charge, and ICG has no net charge with a twitter ion (Scheme 1).

**Figure 1.**
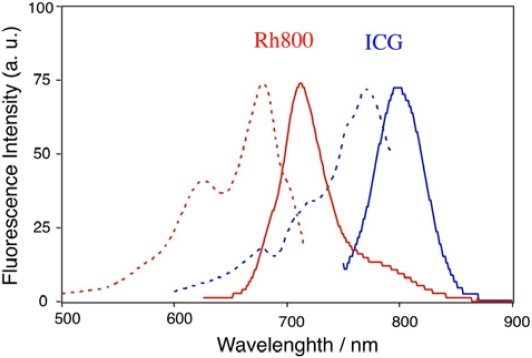
Emission and excitation spectra of Rh800 (red lines) and ICG (blue lines) in PBS. Solid and dotted lines shows emission and excitation spectra, respectively. The concentration of the fluorescent dyes was 40 nM.

Figure 1 shows the emission and excitation spectra of Rh800 and ICG in PBS buffer. Rh800 emits at about 710 nm in PBS. ICG has an emission peak at about 800 nm and can be excited by NIR-light (*circa*700 nm–800 nm). It has been reported that fluorescence life times of Rh800 and ICG are less than 1 ns in aqueous solutions [32,33], and the fluorescence efficiency of these NIR-dyes is much lower than that of fluorescence dyes such as rhodamine 6G and fluorescein in the visible region [30]. Since there are no suitable quantum yield (QY) standards in the NIR region, we used an absolute QY measurement method (see, experimental section) for determining QYs of the NIR-dyes: 3.8 % for Rh800 (in PBS) and 0.5 % for ICG (in water).

### 2.2. The Complexing Abilities of Rh800 and ICG for S[n]

**Figure 2.**
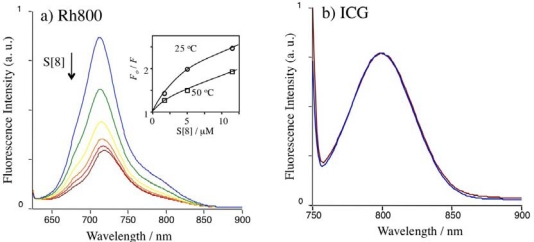
Fluorescence spectra of Rh800 **(a)** and ICG **(b)** upon adding S[8] in PBS. The concentration of Rh800 and ICG was 40 nM. Excitation wavelengths were set to 600 nm for Rh800 and 725 nm for ICG. [S[8]] = 0 μM (blue line), 1.7 μM (green line), 5.0 μM (yellow line), 11.5 μM (orange line), 24 μM (red line), and 52 μM (brown line).

To examine the complexing abilities of Rh800 and ICG, we measured their fluorescence spectra in the presence of S[n]. Since it is well known that S[8] strongly binds to rhodamine 6G [27] and rhodamine B [22], we first examined the effects of S[8] on the fluorescence spectra of Rh800 and ICG. (Figure 2). The fluorescence of Rh800 was quenched by S[8]. However, the fluorescence of Rh800 was not completely diminished even with the addition of excess amounts of S[8]. To gain insight for the quenching mechanism [34] for Rh800, we measured the temperature dependence of the fluorescence quenching by S[8]. The Stern-Volmer plots at 25 °C and 50 °C are shown as an inset in Figure 2a. It should be noted that Rh800 is quenched more effectively at lower temperature. This result indicates that the fluorescence quenching of Rh800 dose not result from the dynamic (collisional) interaction between Rh800 and S[8]. In the case of ICG, fluorescence changes were not observed by addition of S[8], indicating that ICG (as a twitter ion) has no binding ability to S[8]. This result is consistent with our previously reported result [23] that a cationic fluorescent ACh analog, dansylcholine binds to S[8], while a neutral fluorescent ACh analog, dansylsulfoamide does not bind to S[8].

**Figure 3.**
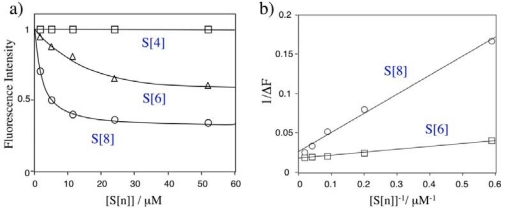
(a) Relative fluorescence intensity of Rh800 (40 nM in PBS) in the presence of S[n]. S[n] (1 mM) was added to the aqueous solution of Rh800 (40 nM) under stirring. (b) Plots of 1/ΔF *versus* 1/ [*S*[n]].

Figure 3 shows changes in the relative fluorescence intensity of Rh800 upon adding S[n]. The fluorescence quenching of Rh800 by S[n] was significantly affected by the size of S[n]. Even at the presence of 1,300 equivalents of S[4], fluorescence intensity of the Rh800 was not changed. In contrast, the addition of S[6] and S[8] significantly quenched the fluorescence intensity of Rh800. The excess amounts of S[6] and S[8] decreased the fluorescence intensity of Rh800 by a factor of 40 % and 70%, respectively. This size dependency suggests that the quenching of Rh800 results from the static interaction (complex formation) between Rh800 and S[n], but not from the collisional interaction between Rh800 and S[n]. Assuming that Rh800 forms a 1:1 complex with S[n], the dissociation constants (*K_d_*) of Rh800-S[n] complex can be rationalized to the fluorescence intensity change (*ΔF*) in the presence of excess amounts of S[n]:

(1) $$\frac{1}{\Delta F}=\frac{1}{c}+\frac{K_{d}}{c[S[n]]}$$

where *c* is a constant. As can be seen from Figure 3b, the plots of *1/ΔF versus [S[n]]* shows linear relationships, indicating that S[n] (n = 6 and 8) forms a 1:1 complex with Rh800. The dissociation constants are determined to be 9.5 μM and 2.2 μM for the Rh800 complex with S[6] and S[8], respectively. This result shows that the binding affinity of Rh800 to S[8] is about four-times larger than that of Rh800 to S[6]. In the case of S[4], the binding affinity of Rh800 to S[4] is very low to determine the dissociation constant.

### 2.3. Fluorescence Detection of ACh Using Rh800-S[8] Complex

**Figure 4.**
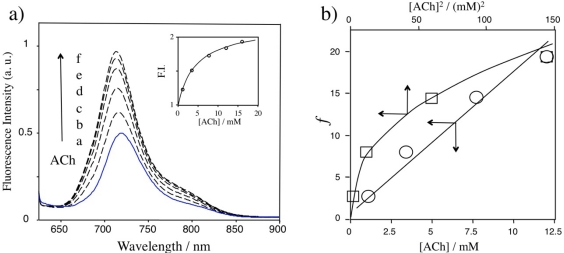
**(a)** Fluorescence spectra of the aqueous solution of Rh800-S[8] complex upon adding ACh. [Rh800] = 40 nM, [S[8]]/[Rh800] = 800, and [ACh]: a) 0 mM, b) 1.1 mM, c) 3.4 mM, d) 7.7 mM, e)12 mM, and f) 16 mM. Inset shows concentration dependence of ACh on the fluorescence intensity of Rh800. **(b)** Plots of *f versus* [ACh] and *f versus* [ACh]^2^.

Figure 4a shows the fluorescence change of Rh800 in the aqueous solution of Rh800-S[8] complex upon adding ACh. The fluorescence intensity of Rh800 significantly increased with increasing ACh concentration. The inset shows the dependence of the relative fluorescence intensity of Rh800 at 710 nm on the ACh concentration. This result shows that the fluorescence changes in the Rh800-S[8] complex solution can be used for the detection of ACh in aqueous solution. The increase in the fluorescence intensity of Rh800 is explained by the competitive displacement of Rh800 by ACh:

(2) [Rh800−S[8]] +n⋅ACh     →    [n⋅ACh−S[8]] +Rh800

In the presence of excess amounts (500 equiv.) of ACh over Rh800, the intensity of Rh800 increased by a factor of 87%. The inset graph indicates that the detection limit (10% fluorescence change) of ACh is *circa* 5 × 10^−4^ M. In the competitive replacement reaction, the dissociation constant (*K_Rh800_*) for the Rh800-S[8] complex can be rationalized to the dissociation constant (*K_ACh_*) for the ACh-S[8] complex as follows:

(3) $$\frac{\alpha ([S[8]]-(1-\alpha )[\mathrm{Rh}800]}{(1-\alpha )K_{\mathrm{Rh}800}}\;\;=\;\;\frac{{\left[\mathrm{ACh}\right]}^{n}}{K_{\mathrm{ACh}}},\;\;\;\;\;\;\;\;\;\;\alpha =\frac{\left(F-F_{o}\right)}{\left(F_{\infty }-F_{o}\right)}$$

where *F_o_, F*, and *F_∞_* are the fluorescence intensity of the Rh800 before, during, and after the titration with ACh in large excess amounts. Figure 4b shows plots of *f (=α([S[8]]-(1−α)[Rh800])/(1−α)K_Rh800_) versus* [ACh] or [ACh]^2^. When *n* is set to 1, the plot shows a linear relationship, indicating that one ACh molecule is replaced by one Rh800 molecule in the Rh800-S[8] complex. From the slope of the plot, the value of *K_ACh_* for the ACh-S[8] complex is determined to be 1.7 mM.

### 2.4. Detection Selectivity of Rh800-S[8] Complex

**Figure 5.**
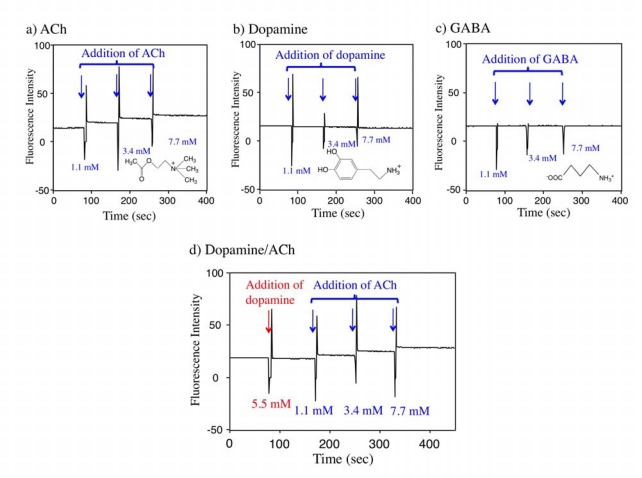
Time course of the fluorescence intensity of Rh800-S[8] solution upon adding (a) ACh, (b) dopamine, and (c) GABA. (d) Changes in the fluorescence intensity of the Rh800-S[8] solution by successive addition of dopamine and ACh. Aliquots of the neurotransmitters (350 mM) were added to the Rh800-S[8] solution. Fluorescence intensity was measured at 710 nm with excitation at 600 nm. [Rh800] = 40 nM, [S[8]]/[Rh800] = 800.

To check the detection selectivity of the Rh800-S[8] complex, we first examined the effects of ACh, dopamine, and GABA on the fluorescence intensity of the Rh800-S[8] complex. Dopamine is one of monoamine neurotransmitters that have cation charges similar to ACh [35]. GABA (γ-aminobutyric acid) is the chief inhibitory neurotransmitter in the central nerve system [36]. GABA is a kind of amino acids with no net charge. We measured the time course of the fluorescence intensity of the Rh800-S[8] solution upon addition of ACh, dopamine, and GABA. The fluorescence intensity of Rh800 responded to the successive additions of ACh as shown in Figure 5a. In contrast, the addition of dopamine and GABA up to 7.7 mM did not change the fluorescence intensity of Rh800 (Figure 5b and 5c). The effect of ACh on the Rh800 fluorescence was also examined in the presence of 5.5 mM of dopamine. As shown in Figure 5d, the fluorescence response of Rh800 for ACh was not hampered by dopamine. These results show that the fluorescence response of Rh800-S[8] complex is sensitive to ACh compared to dopamine and GABA.

**Figure 6.**
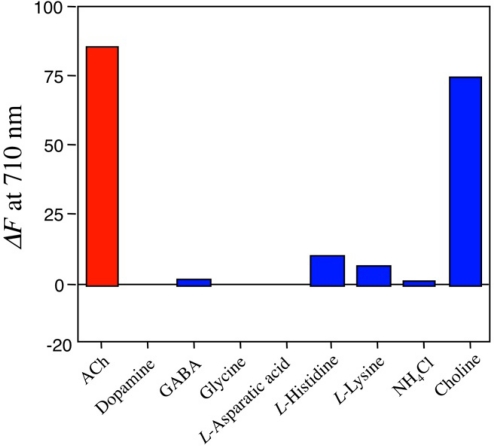
Fluorescence changes (*ΔF* at 710 nm) in Rh800-S[8] solution upon adding ACh, other neurotransmitters (dopamine, GABA, glysine, *l*-asparatic acid), basic amino acids (*l*-histidine, *l*-lysine), ammonium chloride, and choline. 100 μL of these chemicals (350 mM in water) was added to the Rh800-S[8] solution (3 mL), where [Rh800] = 40 nM and [S[8]]/[Rh800] = 800.

We further examined fluorescence responses of the Rh800-S[8] complex for other neurotransmitters (glysine and *l*-asparatic acid), basic amino acids, ammonium chloride, and choline. The results including ACh, dopamine, and GABA are summarized in Figure 6. Among the neurotransmitters examined, ACh was most sensitive to change in the fluorescence intensity of Rh800. In the presence of ACh, the fluorescence intensity was increased by a factor of 87%. Choline also increased the fluorescence intensity by a factor of 70%. Other neurotransmitters such as dopamine, GABA, glycine, and *l*-asparatic acid scarcely affected the fluorescence intensity of Rh800-S[8] solution. Basic amino acids with cationic charges (*l*-histidine and *l*-lysine) slightly increased the fluorescence intensity of the Rh800-S[8] solution, while ammonium chloride did not affect its fluorescence intensity. These results indicate that hydrophobic interaction and electrostatic interaction play important roles in the formation of complex between S[8] and ACh. The strong binding ability of S[8] for choline implies that the quaternary ammonium moieties of ACh and choline are recognized by the π-basic cavity [16,20,23] of S[8].

In the view of biological application, the detection selectivity of ACh over choline is very important because ACh is rapidly hydrolyzed to choline and acetic acids by acetylcholineesterase *in vivo*. Unfortunately, the Rh800-S[8] complex cannot distinguish between ACh and choline. Thus, for using Rh800-S[8] complex as a fluorescent ACh probe in biological samples, it would be necessary to perform microwave deactivation [37] of acetylcholineesterase to prevent the hydrolysis of ACh.

## 3. Experimental Section

### 3.1. Materials

p-Sulfonatocalix[4]arene (S[4]), p-sulfonatocalix[6]arene (S[6]), indocyanine greeen (ICG) were purchased from Tokyo Organic Chemicals (Japan). p-Sulfonatocalix[8]arene (S[8]) was purchased from Dojin Chemicals (Japan). Acetylcholine chloride, dopamine hydrochloride were purchase from Wako Chemicals (Japan). Rhodamine 800 (Rh800) was purchased from Anaspec. Other chemicals used are analytical grade.

### 3.2. Fluorescence Spectra and Quantum Yield Measurements

Fluorescence spectra were measured on a FP-6200 spectrofluorometer (Jasco) using a quarts cuvette (1 cm × 1 cm × 4.5 cm), where excitation wavelength was set to 610 nm for Rh800 and 725 nm for ICG. Emission efficiencies of NIR-dyes in PBS were evaluated by using an absolute quantum yield measurement system (C10027, Hamamatsu Photonic). The absolute quantum yield (*QY*) is defined as *QY = PN_em_/PN_ab_*, where *PN_em_* and *PN_ab_* are the number of emitted and absorbed photons by fluorescence materials. Excitation wavelengths were set to 610 nm for Rh800and 725 nm for ICG. The concentration of the NIR dyes was 1 μM.

### 3.3. Fluorescence Titration of NIR Dyes by S[n]

Rh800 and ICG were dissolved in PBS and their concentrations were set to 40 nM. To 3 mL of the NIR-dye solution, microliter aliquots of aqueous solution f S[n] (1 mM) were added under stirring. After stirring for 2 min, fluorescence spectra of the NIR dye were measured.

### 3.4. Competitive Fluorophore Displacement by ACh in the Complex of R800-S[n]

To 3 mL of Rh800 solution (40 nM/PBS), 100 μL of S[n] (1 mM) was added. To this solution, microliter aliquots of ACh (350 mM) were added under stirring. After 2 min stirring, fluorescence spectra were measured. For the competitive fluorophore displacement by other neurotransmitters, amino acids, ammonium chloride, and choline, similar procedure as the above method was applied.

## 4. Conclusions

In conclusion, we have developed a new ACh detection method using NIR fluorescence of the Rh800-S[8] complex. The competitive fluorophore displacement by ACh in the Rh800-S[8] complex caused significant increase in the NIR fluorescence intensity of Rh800. The detection limit of ACh in this method was about 5 × 10^−4^ M. Fluorescence response of the Rh800-S[8] complex was highly specific to ACh over other neurotransmitters such as dopamine, GABA, glysine, and *l*-asparatic acid. Using the Rh800-S[8] complex as a fluorescent probe, ACh could be detected by NIR-fluorescence at 710 nm. To the best of our knowledge, this is a first report of a NIR-fluorescence detection of ACh in aqueous solution. Unfortunately, our NIR detection system cannot discriminate ACh and choline, because p-sulfonatocalix[8]arene has poor binding selectivity for ACh over choline. In addition, the detection limit (5 × 10^−4^ M) of ACh is not enough to measure ACh concentration in biological samples. Thus, for practical application of our ACh detection method for biological systems, detection selectivity and sensitivity for ACh should be improved.
